# Safety assessment of cyclin-dependent kinase 4/6 inhibitors and comparison of time to adverse events

**DOI:** 10.1371/journal.pone.0336767

**Published:** 2025-11-19

**Authors:** Siqi Zhang, Renmin Zhang, Yingzi Xia, Dingwen Cao

**Affiliations:** 1 Department of Pharmacy, Nanjing Lishui People’s Hospital, Nanjing, China; 2 Department of Medical Oncology, Xiang’an Hospital of Xiamen University, School of Medicine, Xiamen University, Xiamen, China; 3 Department of Radiotherapy, Nanjing Lishui People’s Hospital, Nanjing, China; University of Hawai'i at Manoa, UNITED STATES OF AMERICA

## Abstract

**Background:**

Multiple CDK4/6 inhibitors have been approved for the treatment of HR + /HER2- advanced breast cancer. Nevertheless, there is currently a scarcity of safety reports on CDK4/6 inhibitors within large sample cohorts.

**Methods:**

We employed a disproportionality analysis of the FAERS database to detect safety signals for the three marketed CDK4/6 inhibitors (palbociclib, abemaciclib, and ribociclib). We retrieved pertinent reports from 2004 Q1 to 2023 Q3. Four asymmetric analyses were utilized to assess signals.

**Results:**

A total of 459 positive signals were obtained at the preferred term level (146 positive signals for palbociclib, 68 positive signals for abemaciclib, 245 positive signals for ribociclib). Palbociclib-related adverse events were commonly fatigue, white blood cell count decreased, alopecia. Abemaciclib-related adverse events were commonly diarrhea, decreased appetite, dehydration. Ribociclib-related adverse events were commonly neutropenia, white blood cell count decreased and decreased immune responsiveness. Unexpected adverse events related to palbociclib included hot flush, bone marrow failure. Unexpected adverse events related to abemaciclib included myelosuppression, dehydration, and cystatin C increased. Unexpected adverse events related to ribociclib included decreased immune responsiveness, pleural effusion, atrioventricular conduction time shortened.

**Conclusion:**

Our research corroborates the typical adverse events linked to CDK4/6 inhibitors while highlighting potential safety concerns in their real-world clinical application.

## Introduction

Breast cancer is not only the most prevalent cancer among women but also the primary cause of cancer-related deaths in this demographic [1].Breast cancer can be divided into four subtypes based on receptor status: luminal A-like subtype, luminal B-like subtype, HER2 subtype, and basal-like subtype (triple-negative breast cancer) [2]. Luminal A and B-like subtypes are characterized by being hormone receptor positive (HR), including estrogen receptor (ER) and progesterone receptor (PR), and HER2 negative. These subtypes are the most common types of breast cancer, comprising approximately 80% of cases [3].

For HR+ breast cancer, endocrine therapy is an effective systemic treatment. However, in patients with advanced disease, endocrine therapy alone may not effectively control tumor progression [4]. Cyclin-dependent kinases (CDK) are a class of serine/threonine protein kinases and key enzymes in cell cycle regulation. CDK4/6 inhibitors have the ability to bind to the ATP pocket of CDK4/6, thereby inactivating the cyclin D-CDK4/6 complex and resulting in the dephosphorylation of Rb (retinoblastoma protein), causing cell cycle arrest in the G1 phase [5]. In addition, CDK4/6 inhibitors can enhance glycolysis and oxidative metabolism [6] while depleting reducing agents NADPH and glutathione [7], thereby increasing the apoptosis rate of tumor cells.

In current clinical practice, third-generation CDK inhibitors (mainly including palbociclib, abemaciclib, and ribociclib) have received approval from the FDA for the treatment of HR + /HER2- advanced breast cancer. Although CDK4/6 inhibitors have improved survival in patients with HR + /HER2- advanced breast cancer, they are also associated with some adverse events (AEs). For example, palbociclib and ribociclib have been associated with QT interval prolongation, which carries potential cardiotoxicity [8,9]. Multiple studies have indicated that CDK4/6 inhibitors are linked with an elevated risk of various hematologic adverse events, such as neutropenia, anemia, and thrombocytopenia [10–12]. As the use of CDK4/6 inhibitors in the treatment of HR + /HER2- breast cancer patients becomes more widespread, rare adverse events and long-term safety issues have not yet been systematically reported.

Although pivotal clinical trials have provided valuable information on the safety profile of CDK4/6 inhibitors, they have important limitations. Clinical trials are conducted in carefully selected patient populations with strict eligibility criteria, relatively small sample sizes, and limited follow-up durations, which may underrepresent rare, long-latency, or comorbidity-related adverse events. In contrast, spontaneous reporting systems such as the FDA Adverse Event Reporting System (FAERS) capture real-world data across diverse populations, extended treatment durations, and concomitant therapies. FAERS therefore serves as a complementary tool to clinical trial data, enabling the detection of unexpected or rare adverse events that may not be observed during pre-marketing studies. This justifies the need for a pharmacovigilance analysis to provide a broader perspective on the safety of CDK4/6 inhibitors in routine clinical practice.

The U.S. FDA Adverse Event Reporting System (FAERS) is a publicly accessible database designed to compile adverse event reports submitted by healthcare professionals, consumers, manufacturers, and others [13]. Its primary objective is to monitor and assess the post-market safety of FDA-approved drugs and biologics. The goal of our study is to use the FAERS database to detect and characterize safety signals for the marketed CDK4/6 inhibitors (palbociclib, abemaciclib, and ribociclib), explore unknown adverse events and rare severe adverse events, and investigate potential mechanisms. This research aims to provide reference data for the clinical utilization of CDK4/6 inhibitors and improve their management.

## Methods

### Study design and data source

This pharmacovigilance study uses data from the FDA Adverse Event Reporting System (FAERS) database (https://fis.fda.gov/extensions/FPD-QDE-FAERS/FPD-QDE-FAERS.html) for the period from 2004 to the third quarter of 2023. FAERS collects spontaneous reports of adverse events from around the world. The database includes data on demographics and administrative information (DEMO), drug information (DRUG), indications for use (INDI), adverse events (REAC), patient outcomes (OUTC), sources of reports (RPSR), and the start and end dates of drug usage. Since the FAERS database is publicly accessible and patient records are anonymized, informed consent and ethical review are typically not required.

### Data extraction

**Fig 1:**
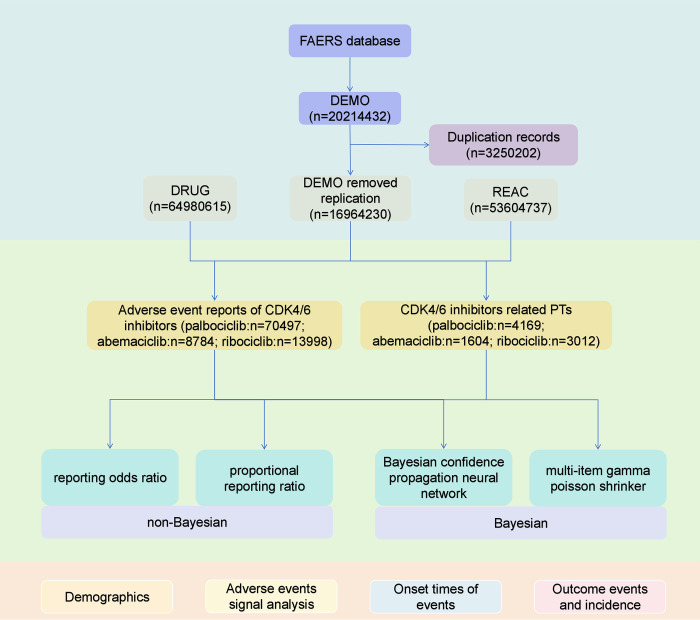
Flow diagram for the selection of AEs associated with CDK4/6 inhibitors.

In FAERS, each reported drug is assigned a role code by the reporter, including primary suspect (PS), secondary suspect (SS), concomitant (C), and interacting (I). For the present analysis, we restricted signal detection to drugs coded as primary suspect (PS), which indicates that the reporter considered the drug most likely responsible for the adverse event. Secondary suspect, concomitant, and interacting drugs were excluded from disproportionality analyses to minimize confounding and ensure that only the most directly implicated drugs were evaluated. Following FDA guidelines, we selected the most recent FDA_DT with the same PRIMARYID and the higher PRIMARYID that matches the FDA_DT and CASEID. This process eliminated duplicate reports submitted by different individuals and institutions. In FAERS, we used the Medical Dictionary for Regulatory Activities (MedDRA) preferred system organ classes (SOC) and preferred terms (PT) [14]. PT is MedDRA’s standard term for uniformly encoding patient-occurring AEs. PTs are unique descriptors of a single medical concept, such as signs and symptoms and disease diagnosis [15]. The names of the three CDK4/6 inhibitors, including generic names and brand names, were used as the standard for fuzzy matching searches in the “drugname” field of the public database of FDA-approved drugs and AEs. The collected data included demographic characteristics and report details. The reported severe outcomes were divided into seven categories: death (DE), life-threatening (LT), hospitalization (initial or prolonged) (HO), disability (DS), congenital anomaly (CA), permanent injury (RI), and other significant medical events (OT). Unexpected AEs were defined as events not included in the official labeling at the time of data extraction. Labeling information was retrieved from the FDA-approved package inserts for palbociclib, ribociclib, and abemaciclib. The process for selecting adverse event reports helps ensure data accuracy and completeness and supports a more in-depth analysis of adverse events related to CDK4/6 inhibitors. Adverse event report selection process **(**Fig 1**)**.

### Signal detection

We used disproportionality analysis to identify drug adverse event signals for CDK4/6 inhibitors [16]. Signal detection was performed using ROR [17], PRR [18], BCPNN [19], and MGPS [20]. The detailed formulas for the four algorithms and the criteria for a positive signal are provided in **(**Table 1 and Table 2**)**. In our study, to avoid false-positive safety signals, an adverse event must meet the positive criteria of all four algorithms simultaneously. The larger the ROR value, the stronger the signal, indicating a more pronounced correlation between the drug and AE. We used R software (version 4.3.1) and Microsoft Excel 2021 for data processing and statistical analysis.

**Table 1:** Four-grid table of signal detection.

Project	Target adverse events	Other adverse events	Total
target drug	a	b	a + b
Other drugs	c	d	c + d
Total	a + c	b + d	N = a + b + c + d

**Notes:** A contingency table for the calculation formula of the disproportionality analysis.

**Table 2:** Four major algorithms used to assess potential associations between CDK4/6 inhibitors and AEs.

Algorithms	Equation	Criteria
ROR	ROR=ad/b/c $\text{95}\%CI={\text{e}}^{\text{ln}(ROR)\pm \text{1}.\text{96}(\text{1}/a+\text{1}/b+\text{1}/c+\text{1}/dhat\;\text{0}.\text{5}}$	lower limit of 95% CI > 1, N ≥ 3
PRR	PRR=a(c+d)/c/(a+b) χ2=[(ad−bc^ 2](a+b+c+d)/[(a+b)(c+d)(a+c)(b+d)]	PRR ≥ 1, χ2 ≥ 4, N ≥ 3
BCPNN	$IC={\text{log}}_{\text{2}}a(a+b+c+d)/((a+c)(a+b))$	IC025 > 0
MGPS	EBGM=a(a+b+c+d)/(a+c)/(a+b) 95%CI=eln(EBGM)±1.96(1/a+1/b+1/c+1/d) ^ 0.5	EBGM05 > 2

Abbreviation: ROR, Reporting Odds Ratio; PRR, Proportional Reporting Ratio; BCPNN, Bayesian Confidence Propagation Neural Network; MGPS, Multi-item Gamma Poisson Shrinker; EBGM, Empirical Bayesian Geometric Mean; CI, Confidence Interval; χ2, Chi-square; IC, Information Component; IC025, the lower limit of the 95% one-sided confidence interval for IC; EBGM05, the lower limit of the 95% CI for EBGM.

## Results

### Descriptive analysis

As of 2023Q1, FAERS has recorded a total of 20,214,432 AE reports, and after removing duplicate reports, accumulated 16,964,230 adverse event reports. This includes 70,497 reports for palbociclib, 8,784 reports for abemaciclib, and 13,998 reports for ribociclib. Detailed demographic and clinical information can be found in **(**Table 3**)**. There has been a yearly increase in adverse events related to CDK4/6 inhibitors since their market launch.

**Table 3:** Baseline data for patients using CDK4/6 inhibitors reported in the FAERS database.

Variables	Palbociclib	Abemaciclib	Ribociclib
Overall	N = 70497	N = 8784	N = 13998
Sex			
Female	66020 (93.6%)	7812 (88.9%)	12788 (91.4%)
Male	1616 (2.3%)	152 (1.7%)	254 (1.8%)
Missing	2861 (4.1%)	820 (9.3%)	956 (6.8%)
Age			
≤17years old	59 (0.1%)	4 (0.0%)	19 (0.1%)
18 ～ 64years old	27092 (38.4%)	2080 (23.7%)	3816 (27.3%)
65 ～ 85years old	31563 (44.8%)	1845 (21.0%)	2560 (18.3%)
≥86years old	2349 (3.3%)	129 (1.5%)	106 (0.8%)
Missing	9434 (13.4%)	4726 (53.8%)	7497 (53.6%)
Reporter countries(Top 5)			
1	United States 57796	United States 6879	United States 5013
2	Argentina 2972	Japan 457	Germany 1123
3	India 2252	China 266	Brazil 525
4	Canada 1238	France 147	India 448
5	Japan 1005	Brazil 131	Colombia 335
Outcomes			
Death	8477(12.02%)	713(8.12%)	2686(19.19%)
Life-threatening	268(0.41%)	89(1.01%)	267(1.91%)
Hospitalization	7703(10.93%)	1574(17.92%)	1987(14.19%)
Disability	110(0.16%)	21(0.24%)	58(0.41%)
Congenital anomaly	4(0.00%)	1(0.01%)	1(0.00%)
Permanent injury	66(0.09%)	15(0.17%)	9(0.06%)
Other	14451(20.50%)	1335(15.20%)	4358(31.13%)
Missing	39400(55.89%)	5036(57.33%)	4632(33.09%)
Get Data Year			
2023	9816	1705	3593
2022	11539	1837	3034
2021	8766	1370	2234
2020	9882	1523	1964
2019	9118	1493	1533

### Signal of preferred terms

In terms of identifying pharmacological risks related to drugs, certain types of PT are considered to have no reference value. After conducting signal screening for adverse event reports related to CDK4/6 inhibitors and excluding adverse events related to product issues, various types of injuries, poisoning and procedural complications, various surgeries and medical operations, various congenital, familial, and genetic diseases, various benign, malignant, or unspecified tumors (including cystic and polyp-like growths), and social environmental factors, a total of 459 positive signals were identified at the preferred term (PT) level: 146 positive signals for palbociclib, 68 positive signals for abemaciclib, and 245 positive signals for ribociclib. These PT reports totaled 74,527 cases: 58,211 cases for palbociclib, 5,800 cases for abemaciclib, and 10,516 cases for ribociclib. The top three most frequent PTs for palbociclib-related adverse events were fatigue, white blood cell count decreased and alopecia. The top three most frequent PTs for abemaciclib-related adverse events were diarrhea, decreased appetite and dehydration. The top three most frequent PTs for ribociclib-related adverse events were neutropenia, white blood cell count decreased and decreased immune responsiveness **(**Table 4**)**.

**Table 4:** The frequency of reports associated with CDK4/6 inhibitors at the preferred terms level.

Palbociclib			Abemaciclib			Ribociclib		
PT	n	ROR	PT	n	ROR	PT	n	ROR
fatigue	12156	4.53 (4.44, 4.61)	diarrhoea	2439	13.73 (13.16, 14.33)	neutropenia	1149	9.01 (8.50, 9.56)
white blood cell count decreased	9432	27.16 (26.57, 27.75)	decreased appetite	324	4.25 (3.80, 4.74)	white blood cell count decreased	914	8.56 (8.01, 9.14)
alopecia	5187	7.25 (7.05, 7.45)	dehydration	280	6.44 (5.73, 7.25)	decreased immune responsiveness	418	50.64 (45.87, 55.92)
neutropenia	3745	8.13 (7.87, 8.40)	white blood cell count decreased	276	8.03 (7.13, 9.04)	disease progression	377	3.33 (3.01, 3.69)
stomatitis	2267	10.90 (10.45, 11.37)	disease progression	234	6.50 (5.72, 7.40)	general physical health deterioration	357	3.39 (3.06, 3.77)
full blood count abnormal	2094	22.60 (21.60, 23.64)	neutropenia	202	4.88 (4.25, 5.60)	electrocardiogram qt prolonged	347	9.63 (8.66, 10.71)
platelet count decreased	1780	4.59 (4.38, 4.81)	anaemia	200	3.20 (2.79, 3.68)	neutrophil count decreased	313	8.18 (7.32, 9.14)
neutrophil count decreased	1700	12.60 (12.00, 13.23)	blood creatinine increased	170	7.86 (6.76, 9.14)	leukopenia	297	6.02 (5.37, 6.75)
disease progression	1693	4.11 (3.92, 4.31)	myelosuppression	146	24.98 (21.21, 29.42)	pleural effusion	285	4.57 (4.06, 5.13)
full blood count decreased	1533	23.86 (22.63, 25.15)	interstitial lung disease	136	9.18 (7.76, 10.87)	illness	264	4.09 (3.62, 4.62)
red blood cell count decreased	1410	14.02 (13.29, 14.80)	platelet count decreased	134	3.92 (3.31, 4.65)	bone pain	255	4.28 (3.78, 4.84)
hot flush	1404	5.54 (5.25, 5.84)	drug intolerance	104	3.50 (2.89, 4.25)	alanine aminotransferase increased	234	3.66 (3.22, 4.16)
epistaxis	1067	3.82 (3.60, 4.06)	pneumonitis	97	12.14 (9.94, 14.83)	hepatic enzyme increased	219	3.38 (2.96, 3.86)
illness	951	4.04 (3.79, 4.31)	hepatic function abnormal	84	7.37 (5.94, 9.13)	aspartate aminotransferase increased	214	3.86 (3.38, 4.42)
bone pain	908	4.18 (3.91, 4.46)	red blood cell count decreased	77	8.35 (6.67, 10.45)	tumour marker increased	174	39.63 (34.03, 46.15)
bone marrow failure	896	11.15 (10.43, 11.93)	neutrophil count decreased	73	5.92 (4.70, 7.45)	red blood cell count decreased	168	5.83 (5.01, 6.79)
leukopenia	820	4.55 (4.25, 4.88)	full blood count decreased	61	9.93 (7.72, 12.77)	liver function test increased	145	7.87 (6.68, 9.27)
hypoacusis	775	4.40 (4.10, 4.73)	hepatotoxicity	50	7.31 (5.54, 9.66)	eating disorder	135	6.17 (5.21, 7.32)
oral pain	649	7.83 (7.24, 8.47)	liver disorder	48	3.45 (2.60, 4.57)	full blood count decreased	128	6.68 (5.61, 7.94)
tumour marker increased	553	38.06 (34.77, 41.65)	full blood count abnormal	46	5.19 (3.89, 6.94)	pneumonitis	119	4.75 (3.96, 5.69)
decreased immune responsiveness	444	14.53 (13.20, 16.00)	taste disorder	43	8.92 (6.61, 12.03)	ascites	115	3.81 (3.17, 4.57)
laboratory test abnormal	435	4.06 (3.69, 4.46)	drug-induced liver injury	40	4.90 (3.59, 6.68)	hepatotoxicity	106	4.96 (4.10, 6.01)
blood test abnormal	430	8.95 (8.13, 9.86)	liver function test increased	38	6.41 (4.66, 8.82)	immunodeficiency	99	8.50 (6.97, 10.36)
white blood cell count abnormal	362	16.72 (15.03, 18.61)	blood potassium decreased	37	3.75 (2.71, 5.18)	spinal pain	99	7.79 (6.39, 9.50)
lacrimation increased	344	3.38 (3.04, 3.76)	hepatic failure	33	3.29 (2.34, 4.63)	lymphocyte count decreased	94	5.21 (4.25, 6.38)
taste disorder	332	6.08 (5.45, 6.78)	tumour marker increased	29	19.86 (13.78, 28.62)	gamma-glutamyltransferase increased	89	3.71 (3.02, 4.58)
myelosuppression	324	4.82 (4.31, 5.38)	eating disorder	23	3.27 (2.17, 4.92)	feeding disorder	89	3.13 (2.54, 3.86)
cytopenia	259	7.46 (6.59, 8.44)	liver injury	22	3.54 (2.33, 5.38)	transaminases increased	84	3.70 (2.99, 4.59)
immune system disorder	259	5.52 (4.88, 6.24)	pneumothorax	22	4.06 (2.67, 6.17)	bone lesion	79	22.06 (17.64, 27.58)
lymphoedema	220	9.16 (8.00, 10.48)	glomerular filtration rate decreased	22	6.42 (4.22, 9.76)	breast pain	78	6.91 (5.53, 8.64)

**Fig 2:**
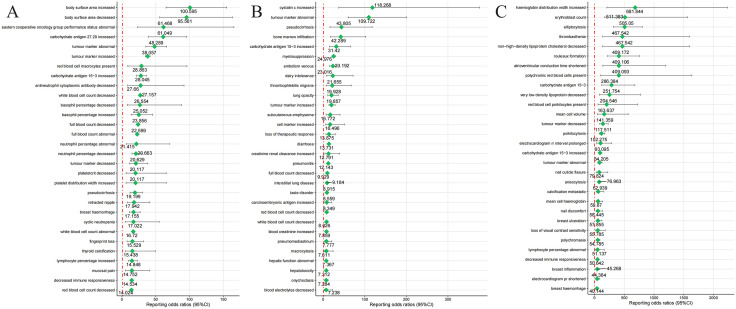
Signal strength of reports associated with CDK4/6 inhibitors at the Preferred Terms level. Notes: Fig 2A describe the top 30 PTs in terms of signal strength for adverse events associated with palbociclib. Fig 2B describe the top 30 PTs in terms of signal strength for adverse events associated with abemaciclib. Fig 2C describe the top 30 PTs in terms of signal strength for adverse events associated with ribociclib.

Further screening of PT signals for CDK4/6 inhibitors showed that palbociclib had high ROR in various examinations **(**Fig 2**)**, with 19 PTs having RORs greater than 20. The top three were body surface area increased, body surface area decreased and Eastern cooperative oncology group performance status abnormal. Abemaciclib had relatively low RORs, with only nine PTs having RORs greater than 20. The top three were cystatin C increased, tumor marker abnormal and pseudocirrhosis. However, ribociclib showed high RORs in various examinations and blood and lymphatic system diseases, with 51 PTs having RORs greater than 20, including 15 PTs with RORs exceeding 100. The top three were hemoglobin distribution width increased, erythroblast count and elliptocytosis.

Additionally, some unexpected adverse events were not mentioned in the drug’s labeling. For instance, unexpected adverse events related to palbociclib included hot flush, bone marrow failure, hypoacusis, antineutrophil cytoplasmic antibody decreased, and carbohydrate antigen 15−3 increased. Unexpected adverse events related to abemaciclib included myelosuppression, dehydration, and cystatin C increased. Unexpected adverse events related to ribociclib included decreased immune responsiveness, pleural effusion, atrioventricular conduction time shortened, elliptocytosis, poikilocytosis, and rouleaux formation.

### Signal of system organ class

**Fig 3:**
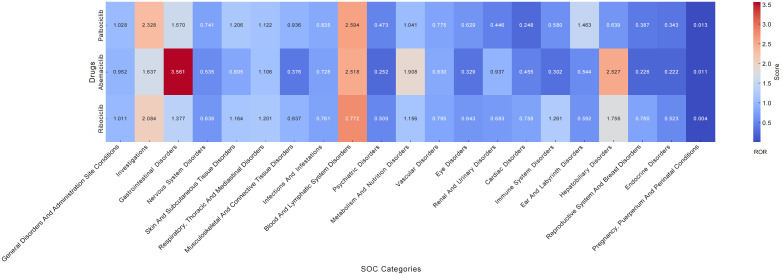
Signal strength of reports associated with CDK4/6 inhibitors at the SOC level.

At the SOC level, adverse events related to CDK4/6 inhibitors involve 27 different SOCs. The three most common systems are general disorders and administration site conditions, investigations, and gastrointestinal disorders. ROR signals for the three CDK4/6 inhibitors **(**Fig 3**)**.

### Time-to-onset analysis

**Fig 4:**
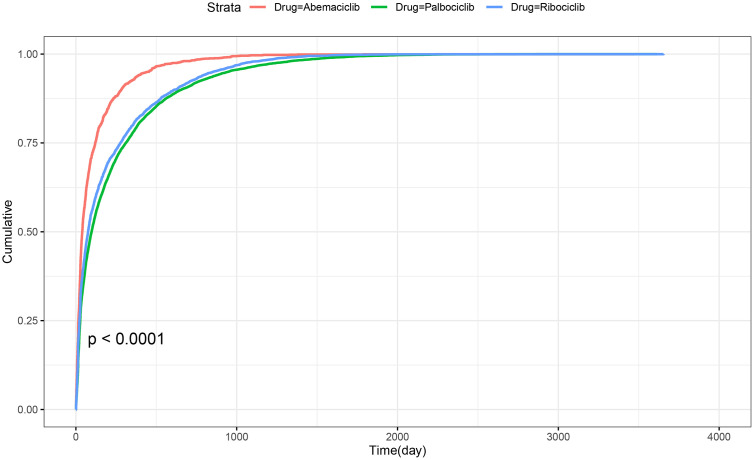
Time to onset of adverse events of CDK4/6 inhibitors.

In terms of the time of occurrence of adverse events, there were a total of 16,983 reports (11,366 for palbociclib, 1,719 for abemaciclib, and 3,898 for ribociclib) that provided information on the timing of the events. Most AEs associated with CDK4/6 inhibitors occurred within the first month of treatment, with 3,235 reports (28.5%) for palbociclib, 738 reports (42.9%) for abemaciclib, and 1,286 reports (33.0%) for ribociclib **(**Fig 4**)**.

## Discussion

CDK4/6 inhibitors effectively suppress tumor growth through multiple pathways, and several agents in this class have been approved by the FDA for treating patients with HR + /HER2- advanced breast cancer. By analyzing FAERS data, our study provides an overview of adverse events (AEs) associated with CDK4/6 inhibitors in real-world practice. These findings can help clinicians better evaluate treatment options and monitor drug safety.

Our results indicate that the most frequent AEs linked to CDK4/6 inhibitors include neutropenia, diarrhea, interstitial lung disease, hepatotoxicity, nausea, vomiting, infections, fatigue, anemia, hair loss, and decreased appetite. These events are consistent with drug labeling information, and several showed strong reporting signals, reflecting their clinical relevance. Importantly, we also identified unexpected AEs not included in drug labels, highlighting the need for further studies to clarify their mechanisms and develop preventive strategies.

All three CDK4/6 inhibitors showed high signal strength for hematologic and lymphatic disorders, including neutropenia, anemia, thrombocytopenia, and reduced red blood cell counts. Palbociclib and ribociclib exhibited stronger hematologic signals than abemaciclib, consistent with previous studies [11,12,21,22]. This difference may be related to variations in drug mechanisms [23,24]. Furthermore, bone marrow-related adverse events, such as bone marrow failure and myelosuppression, were found in the adverse events associated with both palbociclib and abemaciclib. This suggests that in some patients who experience blood cell reduction, there is a need to be particularly vigilant for bone marrow-related adverse events that could worsen the patient’s condition. These findings underscore the critical need for proactive management. For all CDK4/6 inhibitors, we recommend complete blood counts (CBC) prior to the start of therapy, and at the beginning of each subsequent cycle. For patients presenting with profound or persistent cytopenias, particularly those not adequately explained by the typical nadir, clinicians should consider the possibility of underlying bone marrow failure or myelosuppression, and may need to pursue further diagnostic evaluation.

In the gastrointestinal system, diarrhea is the most common adverse event associated with abemaciclib, while palbociclib is more frequently associated with oral mucositis. Compared to the other two drugs, abemaciclib carries a higher risk of gastrointestinal adverse events [25,26]. Its mechanism may be related to off-target effects of abemaciclib; studies have shown that abemaciclib can cause changes in gene expression in rat intestines, with morphological changes such as increased proliferation of crypt cells, loss of goblet cells, poor differentiation and degeneration of enterocytes with loss of microvilli, as well as mucosal inflammation, which are not related to abemaciclib’s inhibitory effects on CDK4/6 [27,28]. Therefore, patients experiencing gastrointestinal adverse events related to abemaciclib should receive symptomatic treatment or consider switching to the other two medications.

With respect to hepatic and renal toxicity, abemaciclib and ribociclib were associated with more frequent laboratory abnormalities, whereas palbociclib appeared relatively safer [29]. Liver system adverse events associated with abemaciclib manifest as abnormal liver function. The mechanism of abemaciclib-induced liver injury is not completely clear; research suggests that abemaciclib-induced hepatotoxicity may be caused by the following pathways: generation of ROS and depletion of GSH; the secretion of pro-inflammatory mediators such as TNF-α and MCP-1 is increased, while the release of the anti-inflammatory cytokine IL-10 is reduced; and increased intracellular calcium levels, inducing early apoptosis and necrotic cell death in HepG2 cells [30]. Kidney system adverse events associated with abemaciclib manifest as increased blood creatinine levels. Previous research indicates that abemaciclib can inhibit tubular secretion without altering glomerular filtration rate [31]. Therefore, when patients experience abnormal liver function or blood creatinine abnormalities, it is important to promptly reduce the dosage or switch to a safer medication.

Among other adverse events, all three CDK4/6 inhibitors have shown neurological system adverse events such as taste disorders, though the underlying mechanism is unclear and requires further research. Additionally, palbociclib has shown a higher occurrence of hearing loss; however, most reports in the data come from individuals aged 65 and older, possibly reflecting degenerative physiological changes. Therefore, physiological factors cannot be excluded from influencing the results. In clinical practice, it is also necessary to monitor patients’ hearing changes and assess whether the association with the medication is strong, and take appropriate measures accordingly. Ribociclib is strongly associated with prolonged electrocardiogram QT intervals. The mechanism of this event is not yet clear, but some studies suggest that ribociclib may cause prolonged electrocardiogram QT intervals by downregulating KCNH2 expression and upregulating SCN5A and SNTA1 expression [32]. Additionally, ribociclib has shown higher frequencies of pleural effusion and decreased immune responsiveness, although the mechanisms are unclear. Therefore, when using ribociclib in clinical practice, monitoring these adverse events and promptly adjusting the treatment plan is essential.

In clinical practice, CDK4/6 inhibitors are almost universally co-administered with endocrine therapies such as aromatase inhibitors or fulvestrant. Many adverse events that we identified are well-established side effects of endocrine therapy. Moreover, patients with advanced or metastatic breast cancer often have comorbidities and disease-related complications that may also contribute to reported adverse events. Because spontaneous reporting systems such as FAERS do not provide detailed treatment sequences, dosage adjustments, or disease burden information, it is impossible to fully disentangle the contributions of CDK4/6 inhibitors versus concomitant endocrine therapy or the underlying malignancy. Therefore, while our findings suggest safety signals warranting attention, these associations should be interpreted with caution, and further prospective or mechanistic studies are needed to clarify causality.

Most AEs occurred within the first month of treatment initiation. This emphasizes the need for intensive monitoring during the early treatment phase, along with continued follow-up during long-term therapy. The time-to-onset analysis was descriptive in nature and did not involve formal survival-based statistical testing. Future studies integrating more complete datasets and applying survival analytic methods will be important to confirm and extend our findings.

The major strength of our study is the use of a large pharmacovigilance database, which allowed us to identify both common and rare AEs. However, several limitations should be acknowledged. First, FAERS data are based on spontaneous reporting, which may lead to underreporting or misclassification, affecting the accuracy of AE incidence [33]. Second, confounding factors such as drug interactions and comorbidities cannot be fully controlled. Third, most reports originate from the Americas, raising concerns about geographic bias. In addition, FAERS data lack detailed clinical information such as precise drug dosage, treatment duration, and patients’ prior therapies. The absence of these data precludes dose-response analysis and limits our ability to fully account for confounding factors, including the effects of concomitant medications or underlying disease severity. Finally, disproportionality analysis cannot establish causality but only suggests potential associations. Prospective clinical studies are therefore needed to validate these findings and further assess the long-term safety and efficacy of CDK4/6 inhibitors.

## Conclusion

This study offers a comprehensive overview of adverse events related to CDK4/6 inhibitors in the treatment of HR + /HER2- advanced breast cancer. By identifying common, unexpected AEs and their onset times, we emphasize the need for vigilant monitoring and personalized treatment strategies to optimize patient outcomes and minimize risks.
